# Characteristics of an optimized patient information material for elderly patients with obstructive pulmonary diseases based on patients’ and experts’ assessment

**DOI:** 10.1186/s40248-017-0087-2

**Published:** 2017-03-14

**Authors:** Gábor Tomisa, Alpár Horváth, Brigitta Dombai, Lilla Tamási

**Affiliations:** 1 Semmelweis University Department of Pulmonology, Diós árok 1/C, 1125 Budapest, Hungary; 2 Chiesi Hungary Ltd., Dunavirág u. 2, 1138 Budapest, Hungary

**Keywords:** COPD, Asthma, Patient education, Therapeutic adherence

## Abstract

**Background:**

Life-long regular use of drugs is necessary in chronic diseases like asthma and COPD. There are several methods to improve adherence including patient information and education; however, their effectiveness on the basis of practical experience is often lower than originally planned and expected. Our objective is to develop a patient information material based on the recommendations of patients and their treating healthcare professionals to fulfill their needs.

**Methods:**

A survey was conducted among pulmonologists (*N* = 262), asthma nurses (*N* = 102), general practitioners (*N* = 321) and patients with obstructive pulmonary disease (*N* = 978) using on line questionnaires.

**Results:**

All surveyed population would prefer to use 1 to 5 pages long, A5 format patient information material based on topics considered important by patients that is appropriately segmented with pictures as well as supplementary information cards adapted to the life situation of patients and the severity of their disease. Questioned population (whose mean age was 57.2) preferred highly informative printed material.

**Conclusions:**

For more effective information and improvement of adherence we recommend newly structured patient information sheets and information cards with content discussed with the targeted patients and their healthcare professionals.

Customized, patient-centered information materials could improve the efficiency of patient education and make the follow-up of the various therapeutic plans easier for patients.

**Electronic supplementary material:**

The online version of this article (doi:10.1186/s40248-017-0087-2) contains supplementary material, which is available to authorized users.

## Background

The number of patients with obstructive pulmonary disease (asthma, COPD) has increased worldwide in the last decade. These patients are not curable but they can be adequately treated, mostly with a long term, sometimes lifelong therapy or therapeutic combinations [1, 2]. Adherence to medication is one of the critical determinants of the successful management of most chronic diseases. Studies, however, repeatedly emphasize that non-adherence to medication is very common (30–70%) [3–5]. In case of inhalative devices the adherence is known to be lower compared to oral therapies, which is the greatest challenge in the current maintenance inhalation therapies [6].

The level of adherence is affected by a number of factors [7], such as patients’ knowledge of their disease, the applied therapy, its duration as well as its expected side effects. Lack of information most likely results in inappropriate use of the device leading to a reduction in the anticipated therapeutic effectiveness which could lead to impaired quality of life of patients, and increased number of exacerbations and mortality on the long term [8, 9].

The ever-growing number of inhalation devices with different instructions of use, varying daily dose schedule and the changes in therapy during years can also significantly increase the risk of non-adherence. Furthermore, the underlying disease may worsen and co-morbidities may appear leading to additional drug therapies [10]. In the light of this we may conclude that it is a great challenge for healthcare professionals (HCP) to provide appropriate information and thus improve patient adherence to prescribed drugs. Patient education is a well-known need: a study in COPD patients defined patient education as an interdisciplinary task of paramount importance [11], several studies observed that there are common errors in inhaler techniques among patients, which can be minimized with proper patient education and regular follow-up [12, 13].

However, the primary authentic source of information for the patients is the treating physician, taking into consideration the different health culture of patients and the limited time available for a HCP to provide information, educate and check the feedback from patients, the role of patient information materials becomes much more significant [14]. Package Leaflets provided in the carton of medicinal products - requirements for the content and format are laid down in specific regulations worldwide including Europe - are specific to a medicine and device and do not provide sufficient information on the disease itself, only describe the indication, posology and method of administration, precautions for use and possible side effects of a medicinal product [15].

Patient information materials may contribute to enhanced individual responsibility and disease awareness, effectiveness of device utilisation as well as the self-management ability of patients [16]. The importance of self-management is also emphasized by international guidelines, as its absence could lead to therapeutic failure and finally to increased mortality [1, 2].

Prior to surveying, our experience with the currently used information materials – with the help of an expert group consisting of pulmonologists – has shown that the quality of these materials is inconsistent and they are not up to date. We reached similar conclusions on the web as well. According to the opinion of the expert group, which is also confirmed by the results of the survey, there is less and less time for professional and appropriately detailed information of patients in the outpatient departments, pulmonary care units and GPs’ practices, all of them dealing with increasing number of patients.

Our objective was to explore and evaluate the level of information patients with asthma and COPD have regarding their disease and drug therapy, as well as the opinion and recommendations of the surveyed groups on the information patients would like to be and should be aware of. As a result, our aim was to compile an optimized patient information material defined by stakeholders.

## Methods

In our study anonymous questionnaires were used to collect data from pulmonologists, asthma nurses, general practitioners as well as patients with obstructive pulmonary disease diagnosed for at least 1 month and coming for follow-up. The aim of the study was to find out the requirements on format and content of an “ideal” patient information material that fulfills the need of both patients and HCPs treating them. Questionnaires were completed by 262 pulmonologists, 102 asthma nurses, 321 GPs, and 978 patients between May and July, 2014. The willingness to provide answer was shown to be very high in pulmonologists (74.8%) and it was also good in asthma nurses (46.4%). The approximately 10% answering rate in GPs significantly differs from the previous two groups but it is acceptable in on line surveys.

Patients’ opinion was surveyed with the assistance of 20 pulmonologists (expert group) working in outpatient departments with high patient numbers. We had 1 month for patient survey. During this period 5 consecutive patients could complete the questionnaire on each day, a total of 978 evaluable patient questionnaires were collected this way. The patients’ willingness to complete the questionnaires and their answers did not affect their medical care in any way.

The design of questionnaires and the number and type of questions for patients and healthcare professionals (HCPs) are summarized in Table 1. For comparison purposes, we tried to ask questions with wording consistent and relevant for both patients and HCPs. Questions are listed in Additional file 1: Annex 1. Hungarian National Authority for Data Protection and Freedom of Information was notified about the data collection and the survey was registered under no.: NAIH-109154.

**Table 1:** Number of questions used in the questionnaire and topics. For detailed questions see Additional file 1: Annex 1

Topic of question	Number of questions			
	Pulmonologist	Asthma nurse	GP	Patient
Examination, administrative and patient education time	3	3	2^a^	-^b^
Deed for patient information material	1	1	1	1
Volume of patient information material	1	1	1	1
Ratio of text/figures	1	1	1	1
Order of preferred information channel	1	1	1	1
The 4 most important topics a patient information material should include	1	1	1	1
The 4 least important topics not necessary to be included in a patient information material	1	1	1	1
Improvement of effectiveness of patient education	1	1	1	-^b^
Need for involvement of a relative of the patient	1	1	1	1
Total number of questions in the questionnaire	11	11	10	7

^a^Due to difference in competency the question on underlying diagnosis could not be used

^b^No relevant question could be used

There were two significant differences in the questionnaires of each group:

1. Pulmonologists and nurses were asked how much time they spend with patients for the first time (including examination, teaching the use of inhalation device, patient education, other administrative tasks) when asthma or COPD is diagnosed, and later when symptomatic or asymptomatic patients come for follow-up. Considering that GPs are not entitled to diagnose either asthma or COPD in the Hungarian healthcare system, this question was deleted from the GP version.
2. For HCPs, we also wanted to know what they think about the methods which could increase the effectiveness of patient education and what they consider as key points considering the wide range of patient information materials currently used, their advantages and disadvantages and their own level of competency and training.
  Results of this survey were analysed with the involvement of altogether 46 pulmonologists in small groups (focus group discussions) conducted 8 times between September and October, 2014. The statistical analysis was conducted using R 3.0.1 software (R Core Team) [17]. Comparisons between groups were performed with analysis of non-parametric test. Fisher’s exact test and chi-square test were used to analyse the two dimensional contingency tables. A value of *P* < 0.05 was considered statistically significant.

## Results

### Patient characteristics

Nine hundred seventy-eight evaluable questionnaires were filled in by patients with obstructive pulmonary disease. Their mean age was 57.2 years (+/−12.05 years). Number of male and female patients was 405 and 573 respectively, 525 of the surveyed patients have been diagnosed with asthma (mean age 54.8 years) and 453 of them with COPD (mean age 59.9 years). There were no significant differences between the asthma and COPD groups regarding their gender or age. It is important to take into account the fact that the majority of surveyed patients belongs to an older age group, which is likely to affect their views and opinions on the questioned topics.

### Time spent with patient education

In the first point of the survey the question we tried to answer was the time doctors and nurses spend with patients during diagnosis and later during follow-up (14). Follow-up of symptomatic and asymptomatic patients was differentiated (Table 2). According to the results, the pulmonologists can spend more than 20 min with a patient only in 14.5% of cases at the time of diagnosis. This rate is lower, even if a symptomatic patient comes for follow-up (11.2%, *P* = 0.29) and significantly lower if the patient coming for follow-up is asymptomatic (2.3%, *P* < 0.001). A GP usually spends less than 10 min with an asymptomatic patient (91.9%) and 10 to 15 min with a symptomatic patient (43.6%). None of the GPs answering the survey can spend 20 min or more with a patient if he/she does not have any symptoms. In general, all surveyed groups spend most of the time with symptomatic patients followed by the diagnosis that includes information on the characteristics of the disease and the first patient education. They can spend the least time with the follow-up of asymptomatic patients.

**Table 2:** Time spent with patients during diagnosis and control investigations

	Time spent with patient^a^ (min)	Pulmonologists (%)	Asthma nurses (%)^b^	GPs (%)
Diagnosis	1–10	16.8	15.7	-
	10–15	42.7	53.0	-
	15–20	26.0	19.6	-
	>20	14.5	11.7	-
Control, Patient without symptoms	1–10	58.4	42.2	91.9
	10–15	34.7	50.9	6.2
	15–20	4.6	6.9	1.9
	>20	2.3	0.0	0.0
Control, patient with symptoms	1–10	7.2	6.9	39.0
	10–15	47.7	46.1	43.6
	15–20	33.9	31.3	13.7
	> 20	11.2	15.7	3.7

^a^ How much time in average they spent with the patient (investigation, demonstration of the correct usage of inhalation devices, education, explanation of the disease, background and future perspectives)

^b^ Regarding the increasing number of patients in the practice - sometimes 30 patients a day or more - the asthma nurses actively participate in the diagnostic process, but the final diagnostic decision is always entitled to the pulmonologist

### Rationale and preferred format of patient information material

90% of the surveyed patients would consider additional patient information materials useful. 96.1% of pulmonologists, 97.1% of nurses and 94.6% of GPs (the difference between patients and HCPs is significant *P* < 0.05) also support the idea of further information materials in addition to the consultation.

Printed patient information material was ranked in the first place by 77.8% of patients, while GPs, pulmonologists and assistants ranked the printed material on the first place at a ratio of 88.5%, 79.8%, and 89.2%, respectively, difference between the two groups (patient-HCP) was significant (*P* = 0.001). Although older generation of HCPs (>50 year) preferred the hard copy format and younger HCPs (< 35 year) favored internet-based materials, this difference was not statistically significant.

At lower rate but all groups indicated internet as a second possible source of getting and providing information while they did not prefer information delivered on other data storage media. Only 18% of patients ranked internet-based information (patient information) on the first place, whereas this rate was 16% amongst HCPs (*P* < 0.57). We may conclude that nearly one-fifth of questioned patients would like to get information primarily from internet, but this need is only noticed by pulmonologists amongst HCPs, all other HCPs underestimate this need of patients.

### Volume of patient information, text-figure ratio

**Fig. 1:**
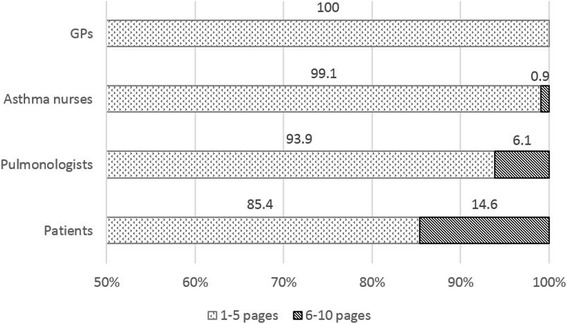
Percentages on the volume of patient information material

According to the vast majority of the surveyed patients (85.4%), patient information materials of 1 to 5 pages (A5 format without cover page) is considered the most effective. It may indicate a significant lack of information (need for information) that 14.6% of patients could accept a patient information material of 6 to 10 pages. However, longer materials are not preferred by either patients or HCPs (Fig. 1).

Based on focus group discussions HCPs would not provide the information materials to their patients as a whole but in 2 parts at the first two visits (typically within 1 month). They considered that segmentation of texts (e.g. by pictures) could contribute to maintain the attention of the patients. In respect of text and figure allocation, significant rate of patients (42.2%) prefers a ratio of 50–50% which is very similar to the opinion of pulmonologists and GPs. Nearly the same rate of patients (42.6%) thought they would prefer more text than figures/pictures in the written patient information.

**Fig. 2:**
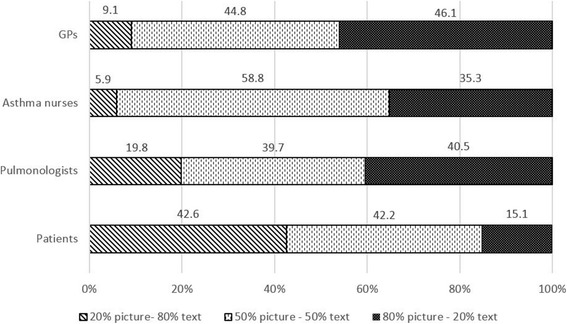
Distribution of picture and text in patient information material as a percentage

Based on the above results it is difficult to define the preference of patients regarding text/figures ratio however, pulmonologists and GPs would more frequently use pictures as demonstration and prefer less informative text in the patient information materials (Fig. 2).

### Key points of patient information materials

Prior to compilation of the questionnaire the expert group of pulmonologist determined the 10 topics they considered patients might be interested in. Patients and HCPs could select the most important ones for them from these topics in our survey.

Table 3 summarizes the responses of patients and healthcare professionals (HCPs) in total and per speciality.

**Table 3:** The full list of topics for patients and HCPs (*HCPs include the opinion of pulmonologists, asthma nurses, and GPs)

Asthma and COPD related topics	Patients (%)	HCPs* (%)	Pulmonologists (%)	Asthma nurses (%)	GPs (%)
Symptoms	10.8	11.7	12.6	11.1	10.6
Causes of disease	13.0	13.0	12.3	13.8	9.0
Triggering and aggravating factors	11.0	12.4	12.8	11.8	12.5
Diagnostic procedure	5.7	4.9	5.2	5.5	3.9
Therapeutic options	20.4	16.8	17.0	16.4	16.9
Alternative therapy	6.6	7.7	7.4	7.5	8.2
Co-morbidities	9.5	4.6	4.0	5.8	3.9
Deterioration of status	7.2	7.4	6.8	6.1	9.2
Lifestyle advice	10.6	17.2	16.7	18.0	17.0
Sports and free time activity	5.2	6.0	5.2	4.0	8.8

The most important topics for patients are in decreasing order of importance: therapeutic options, causes of disease, triggering and aggravating factors, symptoms, lifestyle advice, co-morbidities. Topics are the same for HCPs as well but they are different in terms of rank order. Lifestyle advice is believed much more important by HCPs compared to patients while patients prefer to get more information e.g. on symptoms or other therapeutic options. Taking into consideration that the patient’s needs in terms of the most important topics are not focused on a single subject, to avoid loss of information it was recommended that information cards describing the above topics briefly should be added to the planned patient information material. These cards should be drawn up to shape an integral part of the main patient information material.

Following proposals were given for the content:

- **Topics recommended for patient information for newly diagnosed patients:** symptoms; causes of disease; triggering and aggravating factors; basic therapeutic options; what to do in case of an asthma/COPD attack/exacerbation.
- **Topics recommended for patient information for returning patients:** therapeutic options at advanced level; life style advice; importance of co-morbidities.
- **Information cards:** importance of inhalation steroids (asthma); risks associated with long-term use of inhalation steroids; recognition of exacerbations; importance of rehabilitation (COPD); respiratory exercises; alternative therapies; leisure time and sport; pregnancy/breastfeeding (asthma); importance of diet; allergy and asthma; importance of regular risk assessment; importance of regular use of medicines; authentic web portals, assistance for getting information from internet; patient organisations – patient clubs; customized therapeutic options today and possibilities in the future; importance of rapid - short-acting bronchodilators; role of smoking.
  Patients would always be supplemented by an **individual emergency plan:** treatment in emergency, what to do.

### How to improve the effectiveness of patient information? Is involvement of relatives useful?

In response to the first question a significant number of HCPs (39.6%) indicated that their own education and continued training could bring the most meaningful progress in the improvement in the effectiveness of communication with the patients. Asthma nurses would require continued training at the highest rate (42.2%) followed by GPs at a similar rate (40%) and pulmonologists (38.2%).

58.6% of surveyed patients rejected the involvement of their relatives in the details of treatment, 33.2% welcomed the idea, and 8.2% was not sure about this question. In contrast, most of the HCPs considered the involvement of relatives or at least their information useful, consistently high rate of questioned GPs (61.1%) deemed it necessary and 54.2% of pulmonologists and 54.9% of asthma nurses were of the same opinion.

## Discussion

In our research we - with the involvement of the affected groups (HCPs, patients) - tried to define the characteristics of the ideal patient information material designed mostly for an older patient age group suffering from obstructive pulmonary disease. In the present research, we could aim this patient group based on the average age of the patients who filled in our questionnaire.

While the need for patient education was shown to be extremely high both from patients and medical staff, only a limited time is available for the education and teaching of the use of inhalation device at the diagnosis and control the use later during patients’ follow-up. This is a longstanding, general problem not a country specific one: in fact, several studies conducted so far led to the same conclusion - that errors in inhalation technique and improper therapeutic adherence is a common phenomenon, however proper patient education and regular follow up can improve the situation and help to minimize the errors [12, 13]. With patient information performed in the most cases in harmonized manner, duties are distributed among the involved parties increasing thereby the efficiency of the work of pulmonologists and asthma nurses.

As regard the appropriate format, the preference for printed materials is surprising as state-of-the-art technology has become part of the everyday life of both patients and HCPs by now. It might be explained by the fact that patients may consider that patient information materials provided directly by their doctors are more reliable; additionally, this has been the most known and most frequently used form in the recent decades. Additional benefit includes that doctors can highlight some points in the printed material (important information, topic) on the spot increasing thereby the probability that patients may consider the patient information material as a guidance and useful supplementary information.

The volume of patient information materials was a critical point. It is a challenge to find the optimum volume: important questions may be missing from a too short material leaving uncertainties in the patients; whereas a too long material may result in loss of interest or overlooking of some parts by the patient.

For the ratio of text and figures, it is a cardinal observation that patients - in contrast to the general opinion of doctors and nurses - mostly preferred more informative text content. Healthcare professionals would use pictures and graphical design more than patients. It is an additional interesting conclusion that the more the doctors preferred pictures to text, the more they found a shorter patient information material more reasonable. Therefore, this group would aim to provide simple, user-friendly information. There is another difference in this point between the two groups: GPs and asthma nurses considered the simplest patient information material the best, while pulmonologists generally preferred more complex contents and larger volume which are closer to patients’ requirements.

As GPs meet the patients the most often, it is a surprising result that this group chose the short brochure containing the most pictures at the highest rate; furthermore, a lower rate in this group found the use of patient information material necessary compared to the other groups.

The preferences of the various groups are advisable to be considered for the most important topics to be included in the educational materials. In addition to therapeutic options, patients expressed that they would know more about the cause of disease and the triggering and aggravating factors. Patients would like to have more knowledge and avoid any situations that are associated with exacerbation of the disease. Questioned healthcare professionals (HCPs) had a somewhat different way of thinking and they would better help patients primarily with lifestyle advice focusing on therapeutic options and warning to aggravating factors. While there is a close harmony between patients and medical staff in the selection of important topics, there is a significant difference in viewpoints of two questions. These are lifestyle advice and co-morbidities; the former one is underestimated by patients, and the latter one is underestimated by HCPs compared to the other group. Since patients and doctors found several topics equally important (a ranking can be defined but no significant difference could be detected); therefore, a detailed description of the topics is necessary in an “ideal” patient information material.

**Fig. 3:**
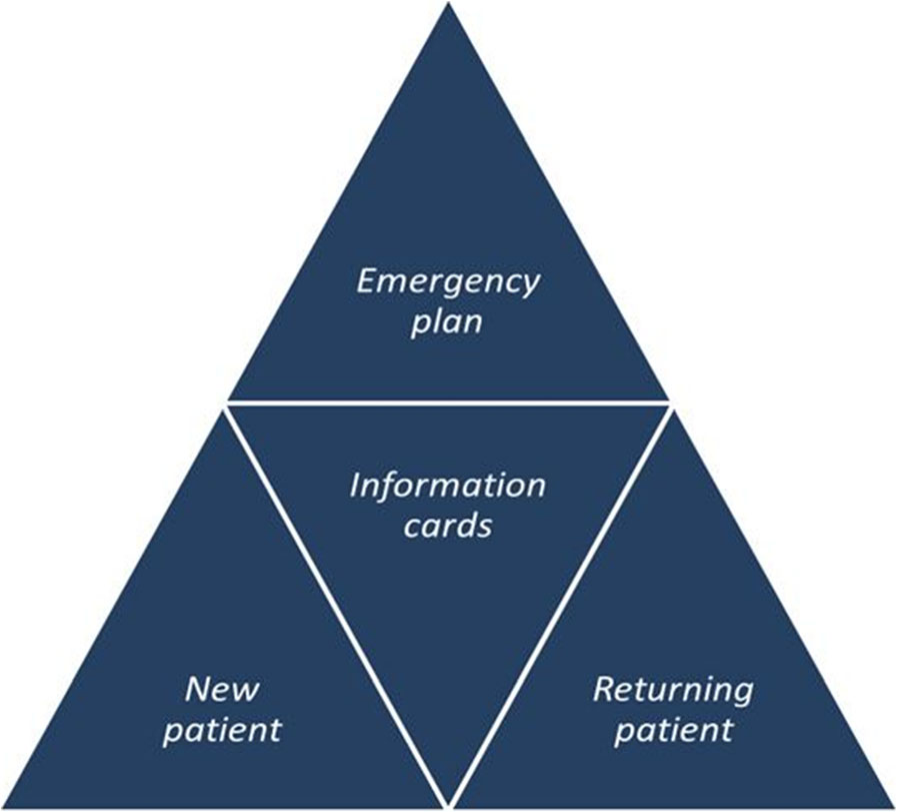
The recommended structure of the patient information material

Based on the results it can be assumed that a structural revision of the materials currently used would be subservient in line with the patients’ requirements. Rather than the conventional patient information brochures, a more flexible and adaptable patient information material series would be advisable that could be customized according to patients’ needs and condition. Not just a new content and format of the patient information material would be required, but a series of publications would also be needed with contents building on and supplementing each other. The basis of these series could be a new type of information materials for “newly diagnosed” patients and another for “returning patients” with information cards and an emergency plan (see Fig. 3).

## Conclusions

A renewed, optimalized, patient-friendly information material, compiled in the structure based on the results of the survey, may result in higher level of information for patients compared to the current options. This would probably improve the exchange of information and communication during doctor-patient visits as a more informed patient could discuss his/her problems with the doctor more efficiently and faster contributing to the improvement of the efficiency of patient care.

With increase in lifespan, number of patients and ratio of chronic illnesses, furthermore considering the limited capacity of healthcare systems we can anticipate that patient information materials based on similar surveys will get into focus worldwide.

Certainly, further studies are necessary to test these optimized information leaflets in the clinical setting. Assessing the patients’ understanding, adherence to treatment and clinical endpoints – such as quality of life, number of exacerbations, mortality – before and after the introduction of these renewed information materials could provide further information about the possible practical benefits.

An important limitation of our study is that the results cannot be generalized to the overall patient population, because the majority of the patients who filled in our questionnaires belong to an older age group. This could influence our results for the reason that elderly patients may find it harder to keep up with the continuous development of technology, therefore they may prefer the conventional, written information materials provided by their doctor. A survey conducted among younger patients most likely would have shown a preference for easy accessible on line content. We would like to further investigate this assumption in the future.

## Additional file

Additional file 1:
**Annex 1.** Questions to patients and HCPs in our research. (DOCX 12 kb)
