# Supplementary material for: Characteristics of an optimized patient information material for elderly patients with obstructive pulmonary diseases based on patients’ and experts’ assessment
Source: Multidiscip Respir Med. 2017 Mar 14;12:6. doi: 10.1186/s40248-017-0087-2 (PMC5351173; doi:10.1186/s40248-017-0087-2)
Supplement: Additional file 1: — Annex 1. Questions to patients and HCPs in our research. (DOCX 12 kb) [file 40248_2017_87_MOESM1_ESM.docx]

**Annex 1** Questions to patients and HCPs in our research.

**Questions for patients:**

1) Do you want to receive patient information material related to your disease from your doctor?

2) What ratio of text and figures would you prefer in the patient information material?

3) Please rank the following forms of patient information. (Printed patient information sheet, Internet-based website, Patient information provided on a data storage media digital data storage)

4) Do you want to involve your relative in questions related to your treatment?

5) How long do you think an effective patient information material should be?

6) What are the 4 most important topics you would like to know in relation to your disease?

7) What are the 4 less important topics you would like to know in relation to your disease?

**Questions for pulmonologists/GPs/asthma nurses:**

1) How much time do you spend with your patient for the first time on average when asthma or COPD is diagnosed? *(not to be used for GPs)*

2) How much time do you spend with a patient on average when he/she comes for follow-up but is asymptomatic?

3) How much time do you spend with a patient on average when he/she comes for follow-up but is symptomatic?

4) In your opinion does your patient want to receive quality patient information material from

his/her doctor?

5) What ratio of text and figures would you prefer in the patient information material?

6) Please rank the following forms of patient information. (printed patient information sheet, Internet-based website, Patient information provided on a data storage media

7) In your opinion does your patient want to involve his/her relative in questions related to his/her treatment?

8) How long do you think an effective patient information material should be?

9) What are the 4 most important topics you would like to know in relation to your disease?

10) What are the 4 less important topics you would like to know in relation to your disease?

11) How do you think the effectiveness of patient education could be further improved?
